# Tandem Approach for Transvenous Lead Extraction: Efficacy, Safety, and Operational Learning Curve

**DOI:** 10.1111/jce.70207

**Published:** 2025-12-08

**Authors:** Alessio Petrone, Zaki Akhtar, Christos Kontogiannis, Viral Sagar, Jaspal Singh Gill, Lisa W.M. Leung, Zia Zuberi, Manav Sohal, Mark M. Gallagher

**Affiliations:** ^1^ Cardiology Clinical Academic Group, City St George's University Hospital NHS Foundation Trust London UK

**Keywords:** Needle's eye femoral snare, operational learning curve, predictors of efficacy and safety, tandem approach, transvenous lead extraction

## Abstract

**Background and Aims:**

The need for transvenous lead extraction (TLE) is rising due to increased cardiovascular device implantation and an aging population. While the superior access is standard, complex cases may benefit from the Tandem approach, combining femoral and superior access to improve efficacy and safety. This study evaluates outcomes and predictors associated with the Tandem approach as a primary strategy.

**Methods:**

A retrospective analysis was conducted on 148 patients who underwent Tandem TLE at a high‐volume UK center between September 2020 and December 2024. Data on procedural success, complete lead removal, complications, and outcome predictors were collected. The Needle's eye snare (NES) learning curve was assessed via fluoroscopy time.

**Results:**

Median patient age was 72.4 years, with 42.6% considered high‐risk (EROS 3). 319 leads were targeted, with 81.2% extracted via the Tandem approach. Clinical procedural success was 97.3%, and complete lead removal 93%. Use of Medtronic leads was the sole independent predictor of complete lead removal. Major complications occurred in 3.4% of cases, with no procedural mortality. BMI < 25 kg/m² and extraction of ≥ 3 leads were predictors of complications and 30‐day mortality. NES proficiency improved significantly after 40 leads (*p* < 0.001), confirming a learning curve.

**Conclusion:**

The Tandem approach is a safe and effective primary strategy for complex TLE, particularly in cases involving passive fixation, shock, and long dwell times leads. However, widespread use may be limited by resource intensity, increased fluoroscopy exposure, and the need for experienced operators.

**Abbreviations**
CIED: cardiovascular implantable electronic devices
NES: Needle's eye snare
RA: right atrium
RV: right ventricle
SVC: superior vena cava
TLE: transvenous lead extraction

## 1 Introduction

The prevalence of transvenous lead extraction (TLE) is steadily increasing, driven by the growing use of cardiovascular implantable electronic devices (CIEDs), an aging population, and expanding indications for extraction [1]. TLE is predominantly performed percutaneously, addressing both infectious and noninfectious indications [2, 3].

However, intravascular and intracardiac fibrosis often complicate the procedure, posing significant challenges and risks [4]. To overcome these difficulties, a range of specialized tools and techniques have been developed [5, 6, 7, 8, 9, 10]. These include locking stylets, rotational sheaths, and snares deployed via femoral or jugular veins [11, 12, 13]. Among these, rotational sheaths, advanced over the lead from the venous entry site, have emerged as the primary tool for lead extraction. While highly effective, these methods are not without risks, including severe morbidity and mortality. Injury to the superior vena cava (SVC) remains the most critical complication, typically resulting from acute angulation at the junction of the innominate vein and SVC where the sheath tip may deviate from a coaxial path, inadvertently advancing into the SVC wall [14, 15].

Despite advancements in technique, achieving complete lead removal from superior access is not always possible. When this occurs, specialized snare tools introduced through the femoral vein are employed to complete the extraction [12, 13]. This “bail‐out” approach is required in approximately 5% of cases, which often involve complex scenarios such as extended lead dwell times or multiple leads [16]. Interestingly, a small group of operators have achieved success rates as high as 98% using femoral access as the primary route for TLE [17].

Additionally, the “Tandem approach”, which combines superior and femoral access, has been introduced as an advanced technique [18, 19]. This method offers geometric advantages and reduces the risk of SVC injury but remains limited to a few specialized centers [20]. At St. George's Hospital, London, UK, a high‐volume center ‐ defined as a center performing over 30 TLE procedures annually—we routinely integrate femoral snare techniques into our initial approach, particularly for high‐risk cases [8].

In this study, we present our pioneering experience with the Tandem approach, employed as a primary procedural step rather than as a “bail‐out” strategy. We analyze its safety and efficacy, assess the predictors of complete lead removal and major complications, and provide insights into the learning curve associated with the Needle's Eye Snare (NES) technique, as performed by a single operator.

## 2 Materials and Methods

We conducted a retrospective analysis of all patients who underwent TLE using the Tandem femoral‐superior technique as the primary approach at St. George's Hospital, between September 2020 and December 2024. A single operator was responsible for utilizing the NES for all cases except those involving the lowest risk targeted leads, while another experienced operator managed the subclavian access.

Patients were stratified into two groups according to the validated ELECTRa Registry Outcome Score (EROS): low risk (EROS 1–2) and high risk (EROS 3) [21]. Patients and procedural data were retrospectively collected using a secure web‐based database, encompassing detailed information on each implanted lead, device, and tool used. The data were analyzed to evaluate the efficacy and safety of the procedure. Specifically, all procedural reports and images were meticulously reviewed to determine clinical procedural success and complete lead removal rates, as well as to document any intraprocedural complications. Fluoroscopy times required to grasp individual leads were recorded to assess the learning curve associated with NES usage. Postprocedure echocardiograms were analyzed to identify and quantify changes in tricuspid regurgitation following the extraction. Progress notes and discharge summaries were reviewed to detect complications arising during the index hospitalization, and to evaluate 30‐day mortality rates (Central Illustration 1).

**Central Illustration 1:**
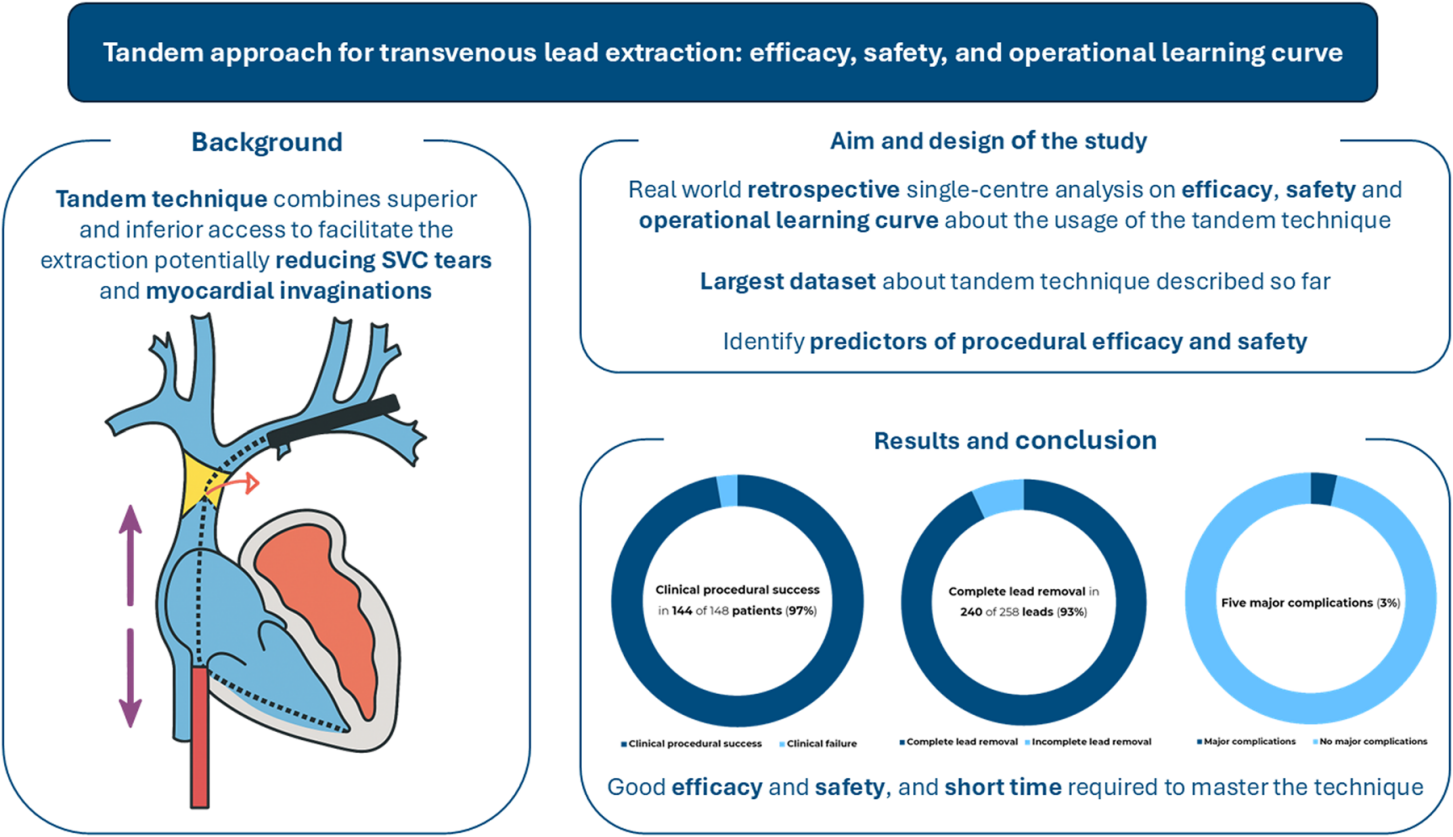
This study presents the largest single‐center retrospective analysis to date on the tandem extraction technique, which leverages both superior and inferior venous access to minimize risks such as superior vena cava (SVC) tears and myocardial invaginations. The research evaluates procedural efficacy, safety, and the associated learning curve, while also identifying predictors of successful and safe outcomes. Results show high procedural efficacy and safety in a high‐risk cohort of patients, with a relatively short time required to master the technique.

The study adhered to local institutional review board guidelines and complied with the principles outlined in the Declaration of Helsinki. All TLE procedures were defined and conducted according to the consensus papers of the Heart Rhythm Society (HRS) and the European Heart Rhythm Association (EHRA) [2, 3].

## 3 Definitions

Outcomes were defined in accordance with HRS and EHRA consensus papers [2, 3].

Clinical procedural success was defined as the removal of all targeted leads without retaining any lead segment longer than 4 cm and without resulting in any permanently disabling complication or procedure‐related death.

Complete lead removal was defined on a per‐lead basis and required no retained lead material visible radiographically.

Complications were defined as any unintended outcomes of the extraction procedure that caused suffering, disability, prolonged hospitalization, or required additional interventions or pharmacological therapy. These complications were further categorized as major or minor. A complication was classified as major if it resulted in the patient's death, persistent disability, or required significant interventions such as cardiac surgery, pericardiocentesis, or vascular surgery. In contrast, a complication was considered minor if it involved an undesirable outcome that did not impair the patient's function or result in death. Procedure‐related fatalities were defined as deaths occurring on the day of the procedure or as a direct consequence of a procedure‐related complication.

## 4 Statistics

Categorical variables were expressed as numbers and percentages, while continuous variables were presented as mean ± standard deviation for normally distributed data, and as median (interquartile range) for nonnormally distributed data. Data distribution was assessed using the Kolmogorov–Smirnov test. Differences between patient groups were analyzed using the *χ*² test for categorical variables and the Student's *t*‐test for continuous variables.

Univariate and multivariate binary logistic regression analyses were conducted to identify predictors of complete lead removal and procedure‐related major complications, including death. Variables that were statistically significant in univariate analysis, along with clinically relevant covariates, were included in the multivariable model. A logarithmic regression analysis was performed to assess the learning curve.

A two‐sided *p*‐value of < 0.05 was considered statistically significant. All statistical analyses were conducted using SPSS Statistics 29 (IBM, Chicago, IL, USA).

## 5 Extraction Technique

For every TLE procedure, a cardiac surgeon and perfusionist were on standby, and the procedure was conducted in the cardiac catheterization suite under general anesthesia, except for one case. Femoral venous access with invasive arterial pressure monitoring was prepared before the extraction, and temporary pacing systems were placed as needed. Standard preoperative imaging included a chest radiograph and transthoracic echocardiogram, with transoesophageal echocardiograms reserved for patients with known or suspected device infections. For each case, blood products were prepared in advance (“Group and Save”), and a SVC occlusion balloon was made available in the Cath lab for immediate use if required in the event of an SVC perforation, but not deployed.

**Figure 1:**
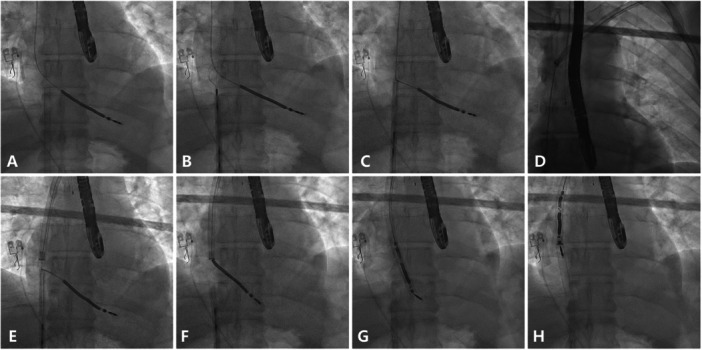
Tandem procedure. (A) At the start of the procedure, the second operator places a 16F outer femoral sheath at the junction between the inferior vena cava and the right atrium (RA). (B) A Needle's eye snare (NES) is advanced through the 12F inner femoral sheath into the RA, positioned around the targeted lead as close as possible to the tricuspid valve. Once the position is satisfactory, the threader is released, and the lead is grasped. (C) After securing the lead, both operators apply traction from top and below in opposite directions to straighten the lead, reducing stress on the superior vena cava (SVC) wall and right ventricular (RV) myocardium during subclavian dissection. (D) Simultaneously, the first operator begins dissection with the rotational sheath from the implant vein. (E) Once the rotational sheath reaches the femoral sheath, the NES is released. (F) With a straight path now cleared, the rotational sheath can advance toward the RV apex, passing only the tricuspid valve. (G) The rotational sheath continues to the RV, working to engulf the lead tip. (H) Once the rotational sheath reaches the distal portion of the lead, the rotating mechanism peels the adhesions away to free the lead tip and complete the extraction.

The Tandem approach procedure followed a standardized protocol (Figure 1 and Video 1). The extraction began with the standard preparation of leads at the implant site. The device pocket was opened and the hardware was dissected free and the leads were mobilized. While the first operator dissected the implant site, the second operator secured femoral venous access and advanced a 16F outer sheath (Merit Medical, UT, USA) through the right femoral vein into the right atrium (RA) (Figure 1A). A curved inner 12F sheath containing the NES was then guided into the RA, where its curved tip improved the direction and reach of the snare compared to a straight inner‐sheath.

For active fixation leads with an extendable–retractable helix, retraction of the helix was attempted by inserting a standard stylet and rotating the proximal pin. If unsuccessful, an attempt to unscrew the lead from the endocardium was made by rotating the snare counterclockwise.

The leads were then cut, and a locking stylet (Liberator Beacon Tip, Merit Medical, UT, USA) was deployed, followed by a compression coil (OneTie, Merit Medical, UT, USA) to secure the lead to the locking stylet. After deploying the locking stylet, the NES was used to grasp the targeted lead in the RA (Figure 1B). Both operators applied firm, opposing traction on the lead to maintain balance, ensuring that the interaction point between the snare and the lead remained in the lower part of the RA. This technique allows to straighten the lead within the SVC and RA (Figure 1C).

A rotational dissecting tool (Evolution RL, Merit Medical, UT, USA; or TightRail, Philips, Amsterdam, Netherlands) was advanced over the lead to dissect it free from adhesions, aided by traction on the locking stylet (Figure 1D). Upon reaching the SVC, the nonrotating outer sheath was advanced in a controlled piston motion to avoid unprotected use of the rotational blade in this region. The rotational sheath was then guided within the outer sheath. Traction and countertraction were maintained while the rotational sheath dissected the lead free from adhesions until it reached the NES (Figure 1E). The lead was released from the snare to allow the rotational sheath to continue toward the lead tip (Figure 1F). When the lead tip was engaged (Figure 1G), the rotational mechanism removed adhesions in a controlled fashion, enabling lead extraction either from above or with assistance from the snare below (Figure 1H).

No active femoral venous closure devices were used after removing the femoral workstation; instead, a figure‐of‐eight stitch was typically applied to the skin and subcutaneous tissues. Postoperatively, patients were placed on 6 h of bed rest.

## 6 Results

### 6.1 Patient and Lead Characteristics

Between September 2020 and December 2024, a total of 148 patients underwent TLE using the Tandem approach as a first‐line strategy. The median patient age was 72.4 years (IQR: 58.5–79.6), and 106 (71.6%) were male. Nearly half the patients, 63 (42.6%), were classified as high‐risk (EROS 3). The most prevalent comorbidities included heart failure (*n* = 83, 56.1%), atrial fibrillation (*n* = 66, 44.6%), hypertension (*n* = 63, 42.6%), ischemic heart disease (*n* = 46, 31.1%), and diabetes (*n* = 36, 24.3%). The average left ventricular ejection fraction (LVEF) was 47.3 ± 13.5%, with a mean CHA_2_DS_2_‐VA score of 3.0.

Device types included dual‐chamber devices in exactly half of the cases (49 pacemakers and 25 defibrillators), with cardiac resynchronization therapy (CRT) accounting for a substantial proportion (13 pacemakers and 30 defibrillators, 29.1% overall). The main indication for TLE was noninfectious (61.5%), predominantly due to lead failure, while infectious indications accounted for the other 38.5% of cases. Additional baseline patient characteristics are summarized in Table 1.

**Table 1:** Baseline characteristics of the patients.

	EROS 1–2 (*n* = 85)	EROS 3 (*n* = 63)	Total (*n* = 148)	*p* value
Age, years, median (IQR)	73.1 (59.3−79.5)	70.2 (58.3−80.2)	72.4 (58.5−79.6)	0.548
Sex				0.289
Male, *n* (%)	58 (68.2)	48 (76.2)	106 (71.6)	
Female, *n* (%)	27 (31.8)	15 (23.8)	42 (28.4)	
BMI, kg/m^2^, median (IQR)	27.1 (23.8−29.6)	26.1 (24.0−30.0)	26.3 (23.9−29.8)	0.605
LVEF (%), mean ± SD	48.4 ± 12.6	45.7 ± 14.4	47.3 ± 13.5	0.232
Heart failure, *n* (%)	52 (61.2)	31 (49.2)	83 (56.1)	0.147
Atrial fibrillation, *n* (%)	36 (42.4)	30 (47.6)	66 (44.6)	0.524
Hypertension, *n* (%)	40 (47.1)	23 (36.5)	63 (42.6)	0.199
Ischemic heart disease, *n* (%)	27 (31.8)	19 (30.2)	46 (31.1)	0.835
Diabetes, *n* (%)	24 (28.2)	12 (19.0)	36 (24.3)	0.198
Chronic kidney disease, *n* (%)	15 (17.6)	13 (20.6)	28 (18.9)	0.646
COPD, *n* (%)	14 (16.5)	10 (15.9)	24 (16.2)	0.922
CHADS‐VA, mean ± SD	3.2 ± 1.9	2.7 ± 1.8	3.0 ± 1.9	0.118
Implanted device				0.134
PM‐SR, *n* (%)	4 (4.7)	8 (12.7)	12 (8.1)	
PM‐DR, *n* (%)	31 (36.5)	18 (28.6)	49 (33.1)	
ICD‐SR, *n* (%)	11 (12.9)	8 (12.7)	19 (12.8)	
ICD‐DR, *n* (%)	10 (11.8)	15 (23.8)	25 (16.9)	
CRT‐P, *n* (%)	9 (10.6)	4 (6.3)	13 (8.8)	
CRT‐D, *n* (%)	20 (23.5)	10 (15.9)	30 (20.3)	
Procedure indication				0.017
Infection, *n* (%)	28 (32.9)	29 (46.0)	57 (38.5)	
Local infection, *n* (%)	21 (24.7)	24 (38.1)	45 (30.4)	
Systemic infection, *n* (%)	7 (8.2)	5 (7.9)	12 (8.1)	
No infection, *n* (%)	57 (67.1)	34 (54.0)	91 (61.5)	
Lead failure, *n* (%)	40 (47.1)	32 (50.8)	72 (48.6)	
Upgrade ± venous occlusion, *n* (%)	17 (20.0)	2 (3.2)	19 (12.8)	

*Note:* Values are presented as *n* (%), mean ± SD, median, and IQR.

Abbreviations: BMI, body mass index; COPD, chronic obstructive pulmonary disease; CRT, cardiac resynchronization therapy; EROS, ELECTRa Registry Outcome Score; ICD, implantable cardioverter defibrillator; IQR, inter‐quartile range; LVEF, left ventricular ejection fraction; PM, pacemaker; SD, standard deviation.

**Figure 2:**
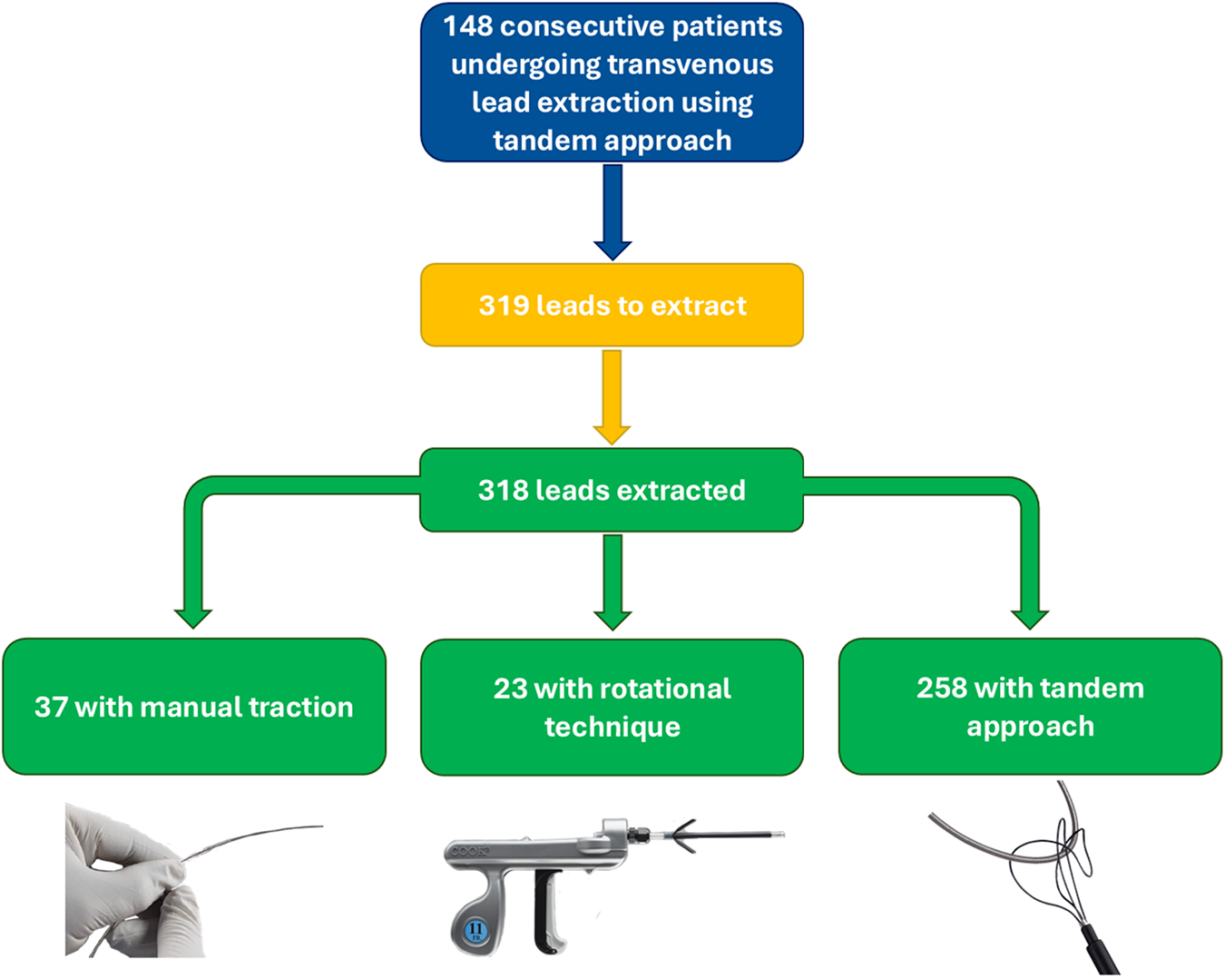
Study flow‐chart.

In total, 319 leads were targeted for extraction (an average of 2.2 leads per procedure), with one lead not extracted due to extensive adhesions and SVC occlusion. Of the remaining 318 leads, 258 (81.2%) were extracted using the Tandem approach, 37 (11.6%) were extracted via manual traction, and 23 (7.2%) using a rotational sheath alone (Figure 2). The primary reason for rotational extraction without tandem assistance was that another lead was spontaneously detached from the heart surface during the procedure, making the Tandem approach unnecessary.

The following analysis focuses exclusively on the 258 leads extracted using the Tandem approach, as they are the primary focus of this study. Among them, the majority were right ventricular (RV) (58.5%), Medtronic (55.0%), pacing (73.3%), and active fixation (62.4%) leads. The mean implant duration was 12.7 ± 7.1 years, with the longest implant duration recorded at 39.9 years. Additional baseline characteristics of the leads are summarized in Table 2.

**Table 2:** Baseline characteristics of the leads.

	Manual traction removal (*n* = 37)	Rotational removal (*n* = 23)	Tandem approach removal (*n* = 258)	All leads (*n* = 318)	*p* value
Dwell time, years, mean ± SD	4.0 ± 3.7	11.7 ± 6.6	12.7 ± 7.1	11.6 ± 7.3	< 0.001
Position					< 0.001
RA, *n* (%)	12 (32.4)	8 (34.8)	92 (35.7)	112 (35.2)	
RV, *n* (%)	13 (35.1)	11 (47.8)	151 (58.5)	175 (55.0)	
LV, *n* (%)	12 (32.4)	4 (17.4)	15 (5.8)	31 (9.7)	
Manufacturer					0.036
Medtronic, *n* (%)	17 (45.9)	12 (52.2)	142 (55.0)	171 (53.8)	
Abbott, *n* (%)	11 (29.7)	3 (13.0)	31 (12.0)	45 (14.2)	
Boston Scientific, *n* (%)	6 (16.2)	5 (21.7)	34 (13.2)	45 (14.2)	
Biotronik, *n* (%)	2 (5.4)	0 (0.0)	24 (9.3)	26 (8.2)	
Microport, *n* (%)	0 (0.0)	1 (4.3)	5 (1.9)	6 (1.9)	
Other, *n* (%)	0 (0.0)	1 (4.3)	4 (1.6)	5 (1.6)	
Unknown, *n* (%)	1 (2.7)	1 (4.3)	18 (7.0)	20 (6.3)	
Type					0.047
Pacing, *n* (%)	34 (91.9)	17 (73.9)	189 (73.3)	240 (75.5)	
Shock, *n* (%)	3 (8.1)	6 (26.1)	69 (26.7)	78 (24.5)	
Fixation					0.538
Active, *n* (%)	24 (64.9)	17 (73.9)	161 (62.4)	202 (63.5)	
Passive, *n* (%)	13 (35.1)	6 (26.1)	97 (37.6)	116 (36.5)	

*Note:* Values are presented as *n* (%) and mean ± SD, median.

Abbreviations: LV, left ventricle; RA, right atrium; RV, right ventricle; SD, standard deviation.

Among all rotational sheaths, the most frequently used was the Evolution RL system (145 cases, 98.0%), while the TightRail was employed in only three cases (2.0%). Specifically, the 13F Evolution RL sheath was utilized in the majority of procedures (*n* = 81, 54.7%). Regarding snares, the NES was used in all procedures, with additional snares (EN Snare or ONE Snare) required in only 27 cases, 19 for retrieval of lead fragments and 8 for the subclavian‐to‐jugular pull‐through technique [10]. Additional equipment characteristics are summarized in Table 3.

**Table 3:** Equipment used.

	Total (*n* = 148)
Snares	
Needle's eye femoral snare, *n* (%)	148 (100)
ONE snare, *n* (%)	23 (15.5)
EN snare, *n* (%)	4 (2.7)
Tools	
One‐tie compression coil, *n* (%)	145 (98.0)
Liberator beacon tip locking stylet, *n* (%)	144 (97.3)
Bulldog lead extender, *n* (%)	30 (20.3)
Rotational sheath	
Evolution shortie RL 9F, *n* (%)	5 (3.4)
Evolution shortie RL 11F, *n* (%)	34 (23.0)
Evolution RL 9F, *n* (%)	21 (14.2)
Evolution RL 11F, *n* (%)	74 (50.0)
Evolution RL 13F, *n* (%)	81 (54.7)
Tightrail, *n* (%)	3 (2.0)

*Note:* Values are presented as *n* (%).

## 7 Efficacy and Safety Outcomes

The median procedure time was 97.0 min (IQR: 68.0−122.5), with a median fluoroscopy time of 937.0 s (IQR: 569.0−1462.0). Clinical procedural success was achieved in 144 of 148 patients (97.3%), with no significant differences according to EROS (*p* = 0.184). Complete lead removal was achieved in 240 of 258 targeted leads (93.0%). The majority of leads (*n* = 292, 89.9%) were removed via the traditional subclavian approach, 8 (3.1%) were extracted using the subclavian‐to‐jugular pull‐through technique, and 18 (7.0%) were completed via the femoral vein access, typically due to lead disruption [10]. Procedural characteristics are summarized in Tables 4 and 5.

**Table 4:** Procedural characteristics and outcomes.

	EROS 1–2 (*n* = 85)	EROS 3 (*n* = 63)	Total (*n* = 148)	*p* value
Procedural characteristics				
Procedure time, minutes, median (IQR)	90.0 (63.0−113.0)	104.0 (84.5−134.0)	97.0 (68.0−122.5)	< 0.001
Fluoroscopy time, seconds, median (IQR)	846.5 (482.0−1311.5)	1100.0 (626.5−1690.0)	937.0 (569.0−1462.0)	0.014
Skin dose, mGy, median (IQR)	37.7 (19.5−60.9)	42.6 (27.4−92.0)	40.1 (22.2−76.9)	0.081
Target leads, mean ± SD	2.0 ± 0.8	2.4 ± 1.0	2.2 ± 0.9	0.014
TPW inserted (pacing dependent), *n* (%)	27 (31.8)	26 (41.3)	53 (35.8)	0.233
Efficacy				0.184
Clinical success, *n* (%)	84 (98.8)	60 (95.2)	144 (97.3)	
Clinical failure, *n* (%)	1 (1.2)	3 (4.8)	4 (2.7)	
Mortality, *n* (%)	1 (1.2)	3 (4.8)	4 (2.7)	0.635
Procedural, *n* (%)	0 (0.0)	0 (0.0)	0 (0.0)	
30‐day, *n* (%)	1 (1.2)	3 (4.8)	4 (2.7)	
Major complications, *n* (%)	2 (2.4)	3 (4.8)	5 (3.4)	0.423
Procedural ventricular fibrillation, *n* (%)	0 (0.0)	2 (3.2)	2 (1.4)	0.257
Severe bradycardia requiring pacing, *n* (%)	1 (1.2)	0 (0.0)	1 (0.7)	0.134
Esophageal perforation, *n* (%)	0 (0.0)	1 (1.6)	1 (0.7)	0.453
Vascular laceration, *n* (%)	1 (1.2)	0 (0.0)	1 (0.7)	0.134
Minor complications, *n* (%)	6 (7.1)	6 (9.5)	12 (8.1)	0.587
Hematoma requiring evacuation, *n* (%)	2 (2.4)	1 (1.6)	3 (2.0)	0.505
Worsening tricuspid valve function, *n* (%)	0 (0.0)	3 (4.8)	3 (2.0)	0.046
Temporary asystole, *n* (%)	1 (1.2)	1 (1.6)	2 (1.4)	1.000
Damage to nontarget leads, *n* (%)	1 (1.2)	0 (0.0)	1 (0.7)	0.296
Pericardial effusion without intervention, *n* (%)	2 (2.4)	0 (0.0)	2 (1.4)	0.121
Hypertensive crisis, *n* (%)	0 (0.0)	1 (1.6)	1 (0.7)	0.296

*Note:* Values are presented as *n* (%), mean ± SD, median and IQR.

Abbreviations: EROS, ELECTRa Registry Outcome Score; IQR, inter‐quartile range; SD, standard deviation; TPW, temporary pacing wire.

**Table 5:** Procedural characteristics of the leads.

	Manual traction removal (*n* = 37)	Rotational removal (*n* = 23)	Tandem approach removal (*n* = 258)	All leads (*n* = 318)	*p* value
Efficacy					0.109
Complete removal, *n* (%)	37 (100)	23 (100)	240 (93.0)	300 (94.3)	
Partial removal, *n* (%)	0 (0.0)	0 (0.0)	18 (7.0)	18 (5.7)	
Removal site					0.160
Subclavian, *n* (%)	37 (100)	23 (100)	232 (89.9)	292 (91.8)	
Jugular, *n* (%)	0 (0.0)	0 (0.0)	8 (3.1)	8 (2.5)	
Femoral, *n* (%)	0 (0.0)	0 (0.0)	18 (7.0)	18 (5.7)	
Tandem approach details					
Grab time, minutes, median (IQR)	/	/	2 (1−5)	/	
Grab fluoroscopy time, seconds, median (IQR)	/	/	32.9 (15.5−81.5)	/	

*Note:* Values are presented as *n* (%), median and IQR.

**Figure 3:**
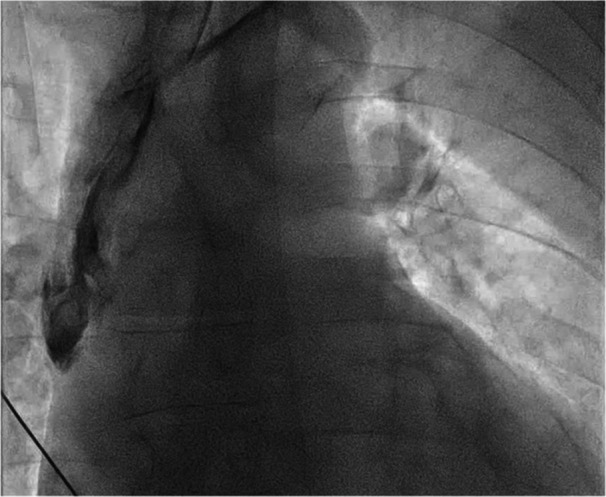
Contrast injection demonstrating a contained SVC dissection originating from the innominate vein.

There were five major complications, with no significant differences according to EROS (*p* = 0.423). Two cases of procedural ventricular fibrillation occurred due to mechanical irritation of the RV during extraction or reimplantation, both successfully terminated with defibrillation and without sequelae. One patient developed symptomatic severe bradycardia the day after the procedure, requiring isoprenaline infusion and permanent pacing. One esophageal perforation occurred, related to the attempt at transoesophageal probe insertion, and unrelated to the extraction itself. Finally, one contained SVC dissection (Figure 3 and Video 2) was observed without hemodynamic compromise, identified during temporary‐permanent pacemaker reimplantation, and managed conservatively. There were no cases of a haemothorax, stroke, or a pericardial effusion requiring drainage and no SVC balloon was deployed. There was no procedural mortality, but four patients (2.7%) died within 30‐days, all of whom presented with systemic infection or poor baseline conditions. Details of all major complications, including 30‐day mortality, are summarized in Table 6.

**Table 6:** Major complications, including 30‐day mortality.

Age	Sex	Indication	Targeted leads	Average dwell time	EROS	Complication	Description
66	M	Pocket infection	5	8.4	3	Death	Death 5 days after the procedure due to mixed shock
6	M	Lead failure	2	10.5	3	Death	Death the day after the procedure due to mixed shock
84	M	Systemic infection	4	10.0	2	Death	Death 20 days after the procedure due to stroke
86	M	Systemic infection	4	20.4	3	Death	Death the day after the procedure due to mixed shock
59	F	Pocket infection	4	19.2	3	Procedural VF	VF while implanting the new RV lead for temporary–permanent pacemaker, requiring 1 x 200J defibrillation with emerging asystole requiring pacing via previously inserted TPW
85	M	Lead failure	4	16.3	3	Procedural VF	Initial hypotensive cardiac arrest requiring CPR, inotropes and fluid resuscitation. Then VF onset requiring 3 x 200J defibrillation to sinus rhythm
87	M	Pocket infection	3	9.1	1	Severe bradycardia	Severe bradycardia requiring isoprenaline infusion and temporary–permanent single‐chamber pacemaker implant
88	M	Pocket infection	2	15.4	3	Esophageal perforation	Perforation during TOE probe insertion
82	M	Pocket infection	3	7.8	1	Vascular laceration	Innominate vein dissection occurred during rotational lead extraction and extended into the SVC during reimplantation when a Terumo guidewire was inadvertently advanced into the false lumen. The complication was managed conservatively, with temporary pacing via the jugular vein (access the true lumen) and successful right‐sided implantation a few days later

Abbreviations: CPR, cardiopulmonary resuscitation; EROS, ELECTRa Registry Outcome Score; F, female; M, male; SVC, superior vena cava; TOE, transoesophageal; TPW, temporary pacing wire; VF, ventricular fibrillation.

Minor complications occurred in 12 out of 148 patients (8.1%), most commonly pocket hematomas requiring evacuation and worsening tricuspid regurgitation. The latter was frequently noted on intraoperative transoesophageal echocardiography but lacked significant clinical correlation. A complete list of outcomes is detailed in Table 4.

### 7.1 Predictors

**Figure 4:**
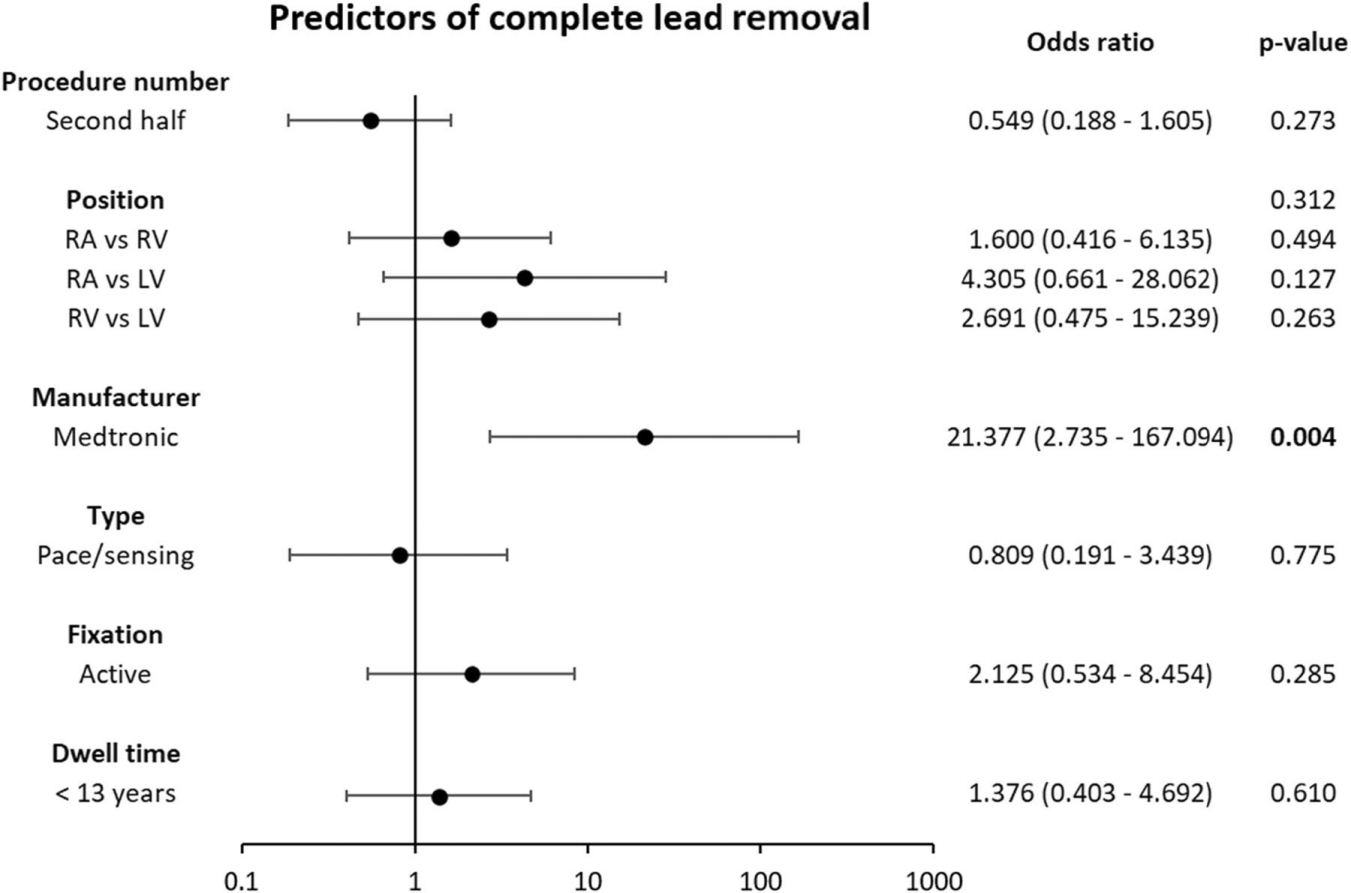
Multivariate analysis on predictors of complete lead removal.

Multivariate logistic regression identified lead manufacturer as the only independent predictor of complete lead removal, with Medtronic leads demonstrating odds of complete removal more than 20 times higher than those of other manufacturers (OR = 21.377, *p* = 0.004) (Figure 4).

**Figure 5:**
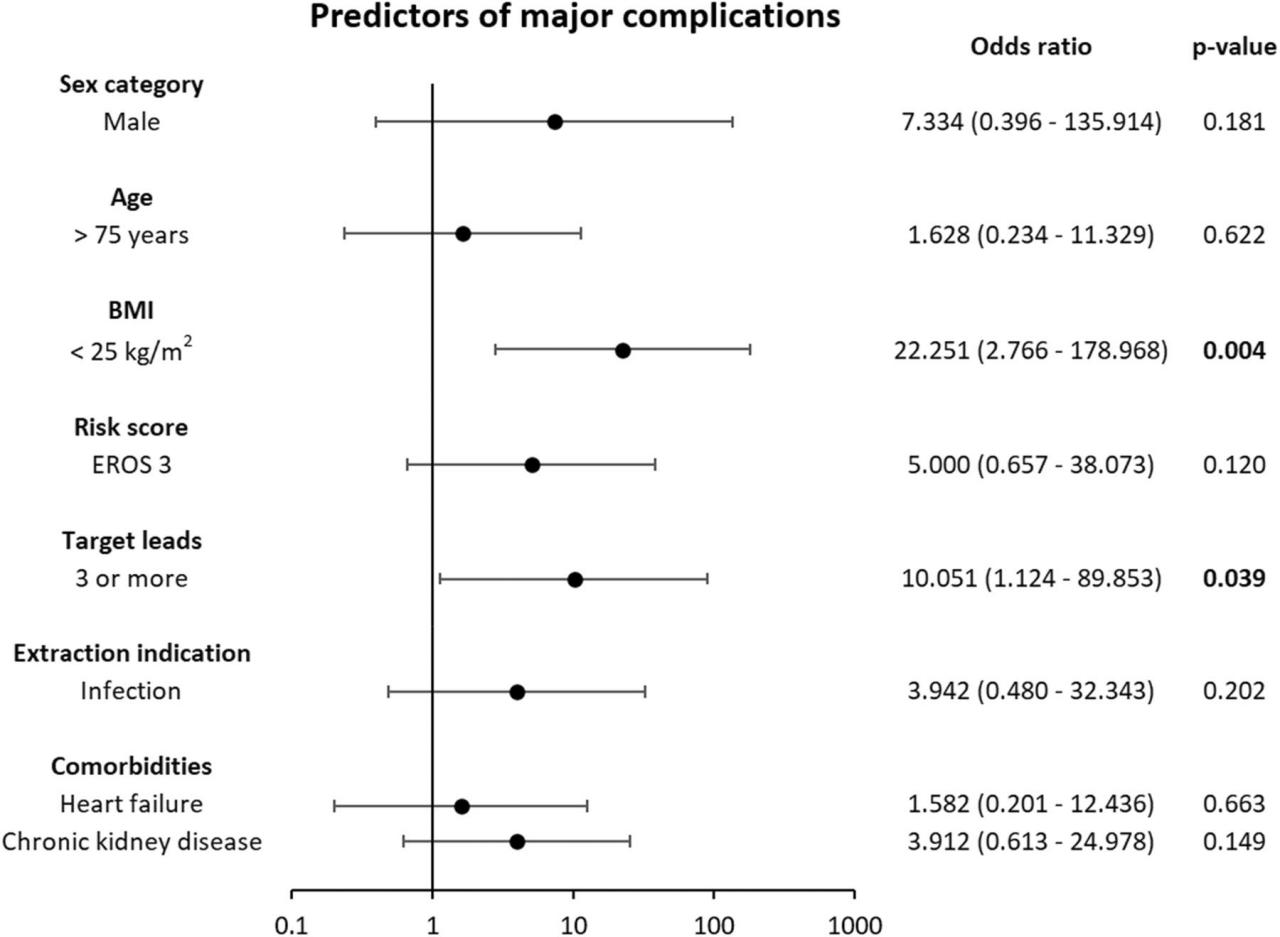
Multivariate analysis on predictors of major complications, including 30‐day mortality.

In the same analysis, predictors of major complications, including 30‐day mortality, were a body mass index (BMI) < 25 kg/m² (OR = 22.251, *p* = 0.004) and procedures involving three or more targeted leads (OR = 10.051, *p* = 0.039) (Figure 5).

### 7.2 Learning Curve

**Figure 6:**
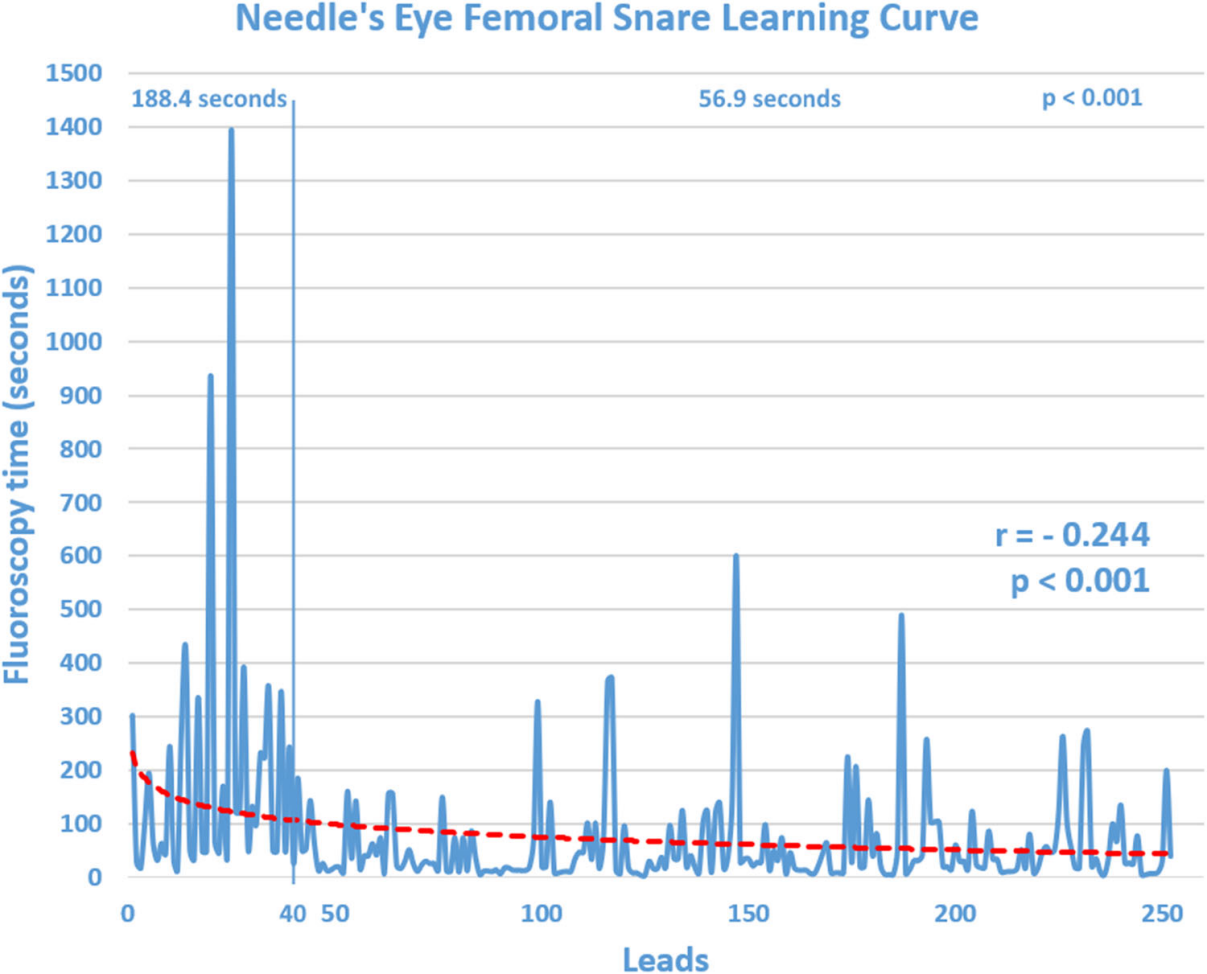
Learning curve for NES usage. The fluoroscopy time required to successfully grasp each lead stabilizes at a plateau after approximately 40 leads.

The median fluoroscopy time required to grasp each lead was 32.9 s (IQR: 15.5−81.5). A statistically significant logarithmic correlation (*r* = −0.244, *p* < 0.001) was observed between fluoroscopy time and the number of leads extracted, with a plateau effect after approximately 40 leads. Indeed, subgroup analysis revealed a notable decrease in fluoroscopy times: the average time to grasp each lead in the first 40 leads was 188.4 s, which significantly decreased to 56.9 s in subsequent cases (*p* < 0.001) (Figure 6).

## 8 Discussion

The Tandem approach evolved from an earlier reliance on a femoral‐only technique for lead extraction and has been described previously [18, 19, 20]. Our study of 148 patients represents the largest reported series of this technique to date. All procedures in our cohort exclusively used a single superior sheath type, a rotational sheath, due to its superior efficacy and safety profile demonstrated in a recent meta‐analysis [15].

The Tandem approach in our series demonstrated efficacy outcomes comparable to those reported in other large TLE studies that predominantly rely on the superior approach, such as ELECTRa and PROMET, where clinical procedural success rates typically range from 96% to 98% [8, 9]. In our cohort, clinical procedural success and complete lead removal rates (97.3% and 93.0%, respectively) aligned closely with the outcomes reported by Muhlestein et al. (96.2% and 92.1%, respectively), who also employed the Tandem approach as a first‐line strategy for TLE [20]. These consistent and reproducible results affirm the effectiveness of the Tandem approach for TLE.

The benefits of the Tandem procedure were particularly evident among patients with leads of prolonged dwell time, passive fixation, and shock leads, categories traditionally associated with higher risks of incomplete lead removal and procedural complications due to dense encapsulation [22, 23, 24, 25, 26, 27]. In our cohort, the average lead dwell time was 12.7 years, the longest reported in any series [5, 7, 8, 9, 20]. Despite this, complete lead removal success was achieved at rates comparable to other studies. Our multivariate logistic regression analysis confirmed that lead dwell time (> 13 years), passive fixation, and shock leads were not predictors of partial lead removal when using the Tandem approach (Figure 4). This success can be attributed to the countertraction provided by the femoral snare, which stretches and straightens the encapsulating tissue, aligning it coaxially with the rotational sheath and enabling a clean and efficient dissection of adhesions. Moreover, the Tandem approach enhances the likelihood of the extraction sheath reaching the lead tip, allowing for more effective and complete dissection of adhesion tissue, which is often most securely bound at this site, particularly in cases of passive fixation [22, 26].

The sole predictor of complete lead removal was the presence of Medtronic leads. These leads are more durable due to their robust design, which resists high mechanical stress. This was confirmed in bench testing, where CapSure Sense and CapSure Fix Medtronic leads demonstrated greater tensile strength than other manufacturers' leads, requiring more force before elongation, especially when snared [28, 29].

An additional advantage of the Tandem approach is the safe extraction of leads that cannot be removed via the superior approach, particularly in cases of SVC occlusion, by using the femoral route with the NES. In our cohort, 18 leads (7.0%) were extracted this way, and only a minority of them required femoral removal due to snare‐lead entanglement.

On the other hand, our results indicate that a Tandem femoral‐superior strategy can achieve comparable complication rates to conventional TLE, with no procedural mortality recorded in our series [2, 3]. A detailed analysis of our five major complications shows that all were reversible, and none were immediately life‐threatening. The single esophageal perforation was related to the attempted transoesophageal probe insertion and not the extraction itself. Two episodes of ventricular fibrillation resulted from guidewire irritation of the RV, a known risk in pacing procedures, and were promptly treated with defibrillation. The severe bradycardia observed the day after the procedure occurred because the patient had been intentionally left without a temporary wire, to allow evaluation of the need for definitive pacing. Finally, the SVC dissection was not directly caused by the rotational tool. In this case, the lead had been successfully extracted using the Tandem approach; during reimplantation, a standard‐length peelable sheath in the innominate vein allowed the new lead to inadvertently enter a dissection plane. When resistance was encountered, a Terumo wire was introduced, which extended the dissection into the SVC due to its high lubricity. Indeed, contrast injection confirmed that the false lumen originated in the innominate vein and extended into the SVC (Figure 3 and Video 2) without consequence. The complication was managed conservatively, with temporary pacing via the jugular vein and successful right‐sided implantation a few days later. This complication highlights an important learning point: following lead extraction, long sheaths should be preferred for reimplantation to bypass potential dissection planes in the venous system instrumented by dissecting sheaths, and ensure safe access to the RA.

Notably, a significant proportion of patients (42.6%) in our series belonged to the EROS 3 risk category, which is reported to be associated with higher complication rates (5.1%) and lower clinical procedural success rates (89%) [21]. Despite this, our efficacy and complication rates remained consistent across all patients, regardless their EROS. This finding is further supported by the multivariate logistic regression, which demonstrated that the EROS 3 risk category was not a predictor of major complications, including 30‐day mortality (Figure 5). These results highlight the potential of the Tandem approach as a first‐line strategy in this high‐risk patient subgroup. Similar outcomes were observed even in patients with other significant risk factors, such as age > 75 years and infectious indications.

One of the most severe complications of TLE is SVC tear, which can result in catastrophic bleeding [30]. SVC damage is believed to occur when the extraction sheath, advanced over the lead from the entry site, is inadvertently directed against the SVC wall during attempts to maneuver it downward within the vessel lumen, particularly at the critical innominate vein‐SVC junction [14]. Additionally, traction from the subclavian site can cause tension transmitted to the heart, determining risks of myocardial avulsion, described as up to 0.6% in the literature [8, 9]. The Tandem approach mitigates the risk of SVC injury by employing a femoral snare to apply traction from below, pulling the lead away from the vessel wall and toward the lumen [31]. This minimizes contact between the rotational sheath and the SVC wall during dissection, thereby reducing the likelihood of injury [20]. Additionally, the femoral snare redirects the point of tension away from the heart during superior traction, transferring it to the snare itself, which significantly lowers the risk of myocardial avulsion [32]. Consistent with these mechanisms, our series observed only one instance of SVC injury, a limited dissection originating from the innominate vein that required no intervention, and no episodes of cardiac avulsions.

The Tandem approach facilitates safe and effective dissection of leads until the dissection sheath reaches the NES in the RA. Beyond this point, the rotational sheath must traverse the tricuspid valve without additional support, maintaining an inherent risk of tricuspid valve injury. As a result, the incidence of such injuries in our series (2%) was consistent with previously published data [2, 3].

Studies have shown that the Tandem approach extraction increases fluoroscopy time [32]. This is expected, as it adds an additional fluoroscopy‐dependent step to the conventional TLE procedure. Visualizing the NES skeleton as it grasps the lead can be challenging, requiring careful attention to the geometry of the interaction between the lead and snare. Achieving proper orientation can be difficult with two‐dimensional fluoroscopy imaging, often necessitating additional projections (particularly left anterior oblique) to identify the correct position for deploying the NES threader; the threader is required to remain anterior to the lead for a successful grasp. This challenge is most pronounced early in the learning curve, when the use of the snare is unfamiliar. However, our data suggest that approximately 40 leads are sufficient to develop expertise in NES usage, after which fluoroscopy dependency significantly decreases.

On the other hand, the overall procedure time tends to decrease with the use of the NES, a secondary benefit of the Tandem approach for several reasons [32]. The opposing traction forces applied to the lead stretch and compress it, improving its ability to detach from binding tissue and reducing overall dissection time. Furthermore, the Tandem technique prepares the “bail‐out” phase from the outset, in contrast to conventional approaches where snaring is typically attempted later, often leading to additional delays. Once proficiency with the NES is achieved, it is not uncommon for the first targeted lead to be grasped even before lead preparation at the implant vein site is completed, saving valuable time, albeit at the cost of increased fluoroscopy usage.

Procedure and fluoroscopy times are not reliable indicators of procedural difficulty and operators' expertise, as they are heavily influenced by various factors, such as the number of targeted leads and the chosen strategy (extraction only or extraction with reimplantation). For this reason, we opted to use individual fluoroscopy times required to grasp each lead as a measure to evaluate the learning curve for NES usage. This approach aimed to provide a more objective assessment, minimizing the impact of confounding variables.

There are significant differences between our study and that of Muhlestein et al., which previously represented the largest cohort of Tandem approach TLE procedures as a first‐line strategy. While both studies involved a similar number of patients and leads, our cohort had a longer average lead dwell time (12.7 years compared to 9.8 years in Muhlestein et al.) [20]. Additionally, our study uniquely analyzed potential predictors of complete lead removal and major complications, including 30‐day mortality, in this group of patients. We also provided novel insights into the learning curve for NES usage, an aspect not previously addressed in the literature.

Despite its benefits, the Tandem approach has inherent limitations. One significant challenge is successfully grasping the lead without disturbing bystander leads that are not targeted for extraction. In some cases, hooking the lead with the NES may be impossible if the lead is tethered to the heart wall with no free segment to engage. Additionally, the femoral approach carries a higher risk of complications. The use of a 16F outer femoral sheath increases the potential for vascular injury, bleeding, and infection [8]. Economic considerations also pose a limitation, as the use of two extraction tools raises the overall cost of the procedure. Moreover, the technique requires two operators working simultaneously, which may not be routinely available in all centers. Consequently, the Tandem approach may be best reserved for complex cases, such as those involving passive fixation, shock leads with prolonged dwell times, especially in older patients with infectious indication, or with an unfavorable anatomy [33]. Conversely, patients with active fixation, pacing leads with short dwell times, and noninfectious indications, particularly younger individuals, may be more appropriately managed with a conventional TLE approach.

## 9 Limitations

This study shares the inherent limitations of observational retrospective designs. However, dedicated data monitors were employed to oversee data entry and ensure accuracy.

Our tandem femoral‐superior strategy was not directly compared with the superior‐only technique. Nonetheless, the baseline patient and lead characteristics in our series are comparable to those in other contemporary lead extraction studies that primarily utilized the superior‐only technique, except for longer implant durations, and consequently higher prevalence of patients with EROS 3 in our cohort.

Additionally, the learning curve for NES usage in this study is based on cases performed by a single operator at a high‐volume center and may not be generalizable to operators in other settings.

The population size was insufficient to detect low‐incidence complications, and the non‐randomized nature of the study presents an important limitation. A randomized study involving multiple operators across various centers would be necessary to reduce the potential influence of technique or experience bias.

## 10 Conclusion

The Tandem procedure can be considered a safe and effective primary technique for TLE, with success rates comparable to those reported in contemporary lead extraction series that predominantly rely on a superior approach. Notably, it can significantly reduce, and potentially eliminate, catastrophic complications such as SVC tears and myocardial avulsions. Furthermore, the learning curve for mastering the Tandem procedure is relatively short, with competency achieved after approximately 40 leads.

However, routine use of this technique is limited by several factors, including increased fluoroscopy exposure, higher economic demands, and the requirement for two operators working simultaneously, which may not be feasible in all centers.

Therefore, the primary advantage of the Tandem approach lies in its suitability for high‐risk extractions, such as cases involving passive fixation, shock leads with long dwell times, especially in older patients with infectious indications for TLE, groups typically associated with worse outcomes.

**Video 1:** Tandem procedure

**Video 2:** Asymptomatic contained SVC dissection originating from the innominate vein

## Conflicts of Interest

The authors declare no conflicts of interest.

## References

[1] D. S. Cannom and E. N. Prystowsky , “The Evolution of the Implantable Cardioverter Defibrillator,” Pacing and Clinical Electrophysiology 27 (2004): 419–431, 10.1111/j.1540-8159.2004.00457.x. 15009880

[2] F. M. Kusumoto , M. H. Schoenfeld , B. L. Wilkoff , et al., “2017 HRS Expert Consensus Statement on Cardiovascular Implantable Electronic Device Lead Management and Extraction,” Heart Rhythm 14, no. 12 (2017): e503–e551, 10.1016/j.hrthm.2017.09.001. 28919379

[3] M. G. Bongiorni , H. Burri , J. C. Deharo , et al., “2018 EHRA Expert Consensus Statement on Lead Extraction: Recommendations on Definitions, Endpoints, Research Trial Design, and Data Collection Requirements for Clinical Scientific Studies and Registries: Endorsed by APHRS/HRS/LAHRS,” EP Europace 20, no. 7 (2018): 1217, 10.1093/europace/euy050. 29566158

[4] J. Keiler , M. Schulze , M. Sombetzki , et al., “Neointimal Fibrotic Lead Encapsulation–Clinical Challenges and Demands for Implantable Cardiac Electronic Devices,” Journal of Cardiology 70, no. 1 (2017): 7–17, 10.1016/j.jjcc.2017.01.011. 28583688

[5] B. L. Wilkoff , C. L. Byrd , C. J. Love , et al., “Pacemaker Lead Extraction With the Laser Sheath: Results of the Pacing Lead Extraction With the Excimer Sheath (PLEXES) Trial11No Financial Support Was Received for Performing the Procedures or Collecting the Data, or for Data Analysis,” Journal of the American College of Cardiology 33, no. 6 (1999): 1671–1676, 10.1016/s0735-1097(99)00074-1. 10334441

[6] M. G. Bongiorni , E. Soldati , G. Zucchelli , et al., “Transvenous Removal of Pacing and Implantable Cardiac Defibrillating Leads Using Single Sheath Mechanical Dilatation and Multiple Venous Approaches: High Success Rate and Safety in More Than 2000 Leads,” European Heart Journal 29, no. 23 (2008): 2886–2893, 10.1093/eurheartj/ehn461. 18948356 PMC2638651

[7] O. Wazni , L. M. Epstein , R. G. Carrillo , et al., “Lead Extraction in the Contemporary Setting: The LExICon Study,” Journal of the American College of Cardiology 55, no. 6 (2010): 579–586, 10.1016/j.jacc.2009.08.070. 20152562

[8] M. G. Bongiorni , C. Kennergren , C. Butter , et al., “The European Lead Extraction ConTRolled (ELECTRa) Study: A European Heart Rhythm Association (EHRA) Registry of Transvenous Lead Extraction Outcomes,” European Heart Journal 38, no. 40 (2017): 2995–3005, 10.1093/eurheartj/ehx080. 28369414

[9] C. T. Starck , E. Gonzalez , O. Al‐Razzo , et al., “Results of the Patient‐Related Outcomes of Mechanical Lead Extraction Techniques (PROMET) Study: A Multicentre Retrospective Study on Advanced Mechanical Lead Extraction Techniques,” EP Europace 22, no. 7 (2020): 1103–1110, 10.1093/europace/euaa103. PMC7336182 32447388

[10] Z. Akhtar and M. M. Gallagher , “Transvenous Lead Extraction: The Subclavian‐to‐Jugular Pull‐Through Technique,” HeartRhythm Case Reports 9, no. 3 (2023): 160–164, 10.1016/j.hrcr.2022.12.001. 36970377 PMC10030300

[11] P. Bordachar , P. Defaye , E. Peyrouse , et al., “Extraction of Old Pacemaker or Cardioverter‐Defibrillator Leads by Laser Sheath Versus Femoral Approach,” Circulation: Arrhythmia and Electrophysiology 3, no. 4 (2010): 319–323, 10.1161/CIRCEP.109.933051. 20562442

[12] L. Epstein and M. Maytin , “Strategies for Transvenous Lead Extraction Procedures,” Journal of Innovations in Cardiac Rhythm Management 8, no. 5 (2017): 2702–2716, 10.19102/icrm.2017.080502. 32494448 PMC7252922

[13] M. F. El‐Chami and F. M. Merchant , “Femoral Extraction of Transvenous Leads and Leadless Pacemakers ‐ A Review of the Data, Tools, and Procedural Steps,” Pacing and Clinical Electrophysiology 42, no. 9 (2019): 1248–1252, 10.1111/pace.13766. 31355937

[14] Y. Arora , L. D'Angelo , R. Azarrafiy , J. Bashir , C. Kennergren , and R. Carrillo , “Location of Superior Vena Cava Tears in Transvenous Lead Extraction,” Annals of Thoracic Surgery 113, no. 4 (2022): 1165–1171, 10.1016/j.athoracsur.2021.04.068. 33964252

[15] Z. Akhtar , C. Kontogiannis , G. Georgiopoulos , et al., “Comparison of Non‐Laser and Laser Transvenous Lead Extraction: A Systematic Review and Meta‐Analysis,” Europace: European Pacing, Arrhythmias, and Cardiac Electrophysiology 25, no. 11 (2023): 1–13, 10.1093/europace/euad316. PMC10638006 37882609

[16] M. F. El‐Chami , F. M. Merchant , A. Waheed , et al., “Predictors and Outcomes of Lead Extraction Requiring a Bailout Femoral Approach: Data From 2 High‐Volume Centers,” Heart Rhythm 14, no. 4 (2017): 548–552, 10.1016/j.hrthm.2017.01.029. 28189825

[17] F. A. Bracke , L. Dekker , and B. M. Van Gelder , “The Needle's Eye Snare as a Primary Tool for Pacing Lead Extraction,” EP Europace 15, no. 7 (2013): 1007–1012, 10.1093/europace/eus426. 23277531

[18] A. Fischer , B. Love , R. Hansalia , and D. Mehta , “Transfemoral Snaring and Stabilization of Pacemaker and Defibrillator Leads to Maintain Vascular Access During Lead Extraction,” Pacing and Clinical Electrophysiology 32 (2009): 336–339, 10.1111/j.1540-8159.2008.02241.x. 19272063

[19] R. D. Schaller , M. M. Sadek , and J. M. Cooper , “Simultaneous Lead Traction From Above and Below: A Novel Technique to Reduce the Risk of Superior Vena Cava Injury During Transvenous Lead Extraction,” Heart Rhythm 15, no. 11 (2018): 1655–1663, 10.1016/j.hrthm.2018.05.022. 29803849

[20] J. B. Muhlestein , E. Dranow , J. Chaney , L. Navaravong , B. A. Steinberg , and R. A. Freedman , “Successful Avoidance of Superior Vena Cava Injury During Transvenous Lead Extraction Using a Tandem Femoral‐Superior Approach,” Heart Rhythm 19, no. 7 (2022): 1104–1108, 10.1016/j.hrthm.2022.02.024. 35245690 PMC9250613

[21] B. S. Sidhu , S. Ayis , J. Gould , et al., “Risk Stratification of Patients Undergoing Transvenous Lead Extraction With the ELECTRa Registry Outcome Score (EROS): An ESC EHRA EORP European Lead Extraction Controlled Electra Registry Analysis,” EP Europace 23, no. 9 (2021): 1462–1471, 10.1093/europace/euab037. 33615342

[22] K. Stokes , J. Anderson , R. McVenes , and C. McClay , “The Encapsulation of Polyurethane‐Insulated Transvenous Cardiac Pacemaker Leads,” Cardiovascular Pathology 4, no. 3 (1995): 163–171, 10.1016/1054-8807(95)00023-x. 25851004

[23] L. Segreti , A. Di Cori , E. Soldati , et al., “Major Predictors of Fibrous Adherences in Transvenous Implantable Cardioverter‐Defibrillator Lead Extraction,” Heart Rhythm 11, no. 12 (2014): 2196–2201, 10.1016/j.hrthm.2014.08.011. 25111324

[24] J. Morita , K. Yamaji , M. Nagashima , et al., “Predictors of Lead Break During Transvenous Lead Extraction,” Journal of Arrhythmia 37, no. 3 (2021): 645–652, 10.1002/joa3.12524. 34141017 PMC8207345

[25] Z. Akhtar , A. I. Elbatran , C. T. Starck , et al., “Transvenous Lead Extraction: The Influence of Age on Patient Outcomes In the PROMET Study Cohort,” Pacing and Clinical Electrophysiology 44, no. 9 (2021): 1540–1548, 10.1111/pace.14310. 34235772

[26] N. Levi , M. G. Bongiorni , M. Rav Acha , et al., “Lead Fixation Mechanism Impacts Outcome of Transvenous Lead Extraction: Data From the European Lead Extraction Controlled Registry,” EP Europace 24, no. 5 (2022): 817–827, 10.1093/europace/euab240. 34652415

[27] A. Ząbek , K. Boczar , M. Ulman , et al., “Mechanical Extraction of Implantable Cardioverter‐Defibrillator Leads With a Dwell Time of More Than 10 Years: Insights From a Single High‐Volume Centre,” EP Europace 25, no. 3 (2023): 1100–1109, 10.1093/europace/euac272. PMC10062326 36660771

[28] C. W. Chan , L. K. Chan , T. Lam , K. K. Tsang , and K. W. Chan , “Comparative Study About the Tensile Strength and Yielding Mechanism of Pacing Lead Among Major Manufacturers,” Pacing and Clinical Electrophysiology 41, no. 7 (2018): 828–833, 10.1111/pace.13376. 29758585

[29] J. Morita , A. Okada , F. Kusumoto , and K. Nakamura , “Comparative Durability of Pacemaker Leads in Transvenous Lead Extraction: An Evaluation Through Bench Testing,” Heart Rhythm O2 6, no. 4 (2025): 481–488, 10.1016/j.hroo.2025.01.015. 40321740 PMC12047550

[30] C. L. Diaz , X. Guo , I. R. Whitman , et al., “Reported Mortality With Rotating Sheaths vs. Laser Sheaths for Transvenous Lead Extraction,” EP Europace 21, no. 11 (2019): 1703–1709, 10.1093/europace/euz238. 31545350

[31] M. Toloui , M. Marshall , P. Vatterott , et al., “Numerical Modeling of Vascular Stresses During Lead Extraction ‐ Subclavian vs. Femoral,” American Society of Mechanical Engineers 1, no. 1 (2020): 1–6, 10.1115/DMD2020-9003.

[32] Z. Akhtar , C. Kontogiannis , A. I. Elbatran , et al., “Transvenous Lead Extraction: Experience of the Tandem Approach,” Europace: European Pacing, Arrhythmias, and Cardiac Electrophysiology 25, no. 11 (2023): 1–9, 10.1093/europace/euad331. PMC10903175 37936325

[33] Z. Akhtar , M. Sohal , C. T. Starck , et al., “Persistent Left Superior Vena Cava Transvenous Lead Extraction: A European Experience,” Journal of Cardiovascular Electrophysiology 33, no. 1 (2022): 102–108, 10.1111/jce.15290. 34783107

